# Concordance between Hopkins Symptom Checklist (HSCL-10) and Pakistan Anxiety and Depression Questionnaire (PADQ), in a rural self-motivated population in Pakistan

**DOI:** 10.1186/1471-244X-8-59

**Published:** 2008-07-22

**Authors:** Hammad Raza Syed, Henrik Daae Zachrisson, Odd Steffen Dalgard, Ingvild Dalen, Nora Ahlberg

**Affiliations:** 1 Institute of General Practice and Community Medicine, Oslo, Norway; 2 Division of Mental Health, Norwegian Institute of Public Health, Oslo, Norway; 3 Institute of Basic Medical Sciences, Department of Biostatistics, University of Oslo, Norway; 4 National Centre for Minority Health Research, Oslo, Norway

## Abstract

**Background:**

There have been no previous studies conducted in Pakistan comparing the concordance of any well established Western anxiety/depression screening instrument with an indigenous scale, in a community based setting.

**Methods:**

Participants (n = 1040) in the present study were recruited from the six villages of our interest from the district Gujarat of Pakistan, using a convenient sampling technique. Interview versions of the Hopkins Symptom Checklist 10-item version (HSCL-10) and the Pakistani Anxiety and Depression Questionnaire (PADQ) were used to observe the pattern of anxiety and depression among the participants.

**Results:**

The internal consistency of HSCL-10 and PADQ were 0.86 and 0.84 respectively. Exploratory factor analysis found evidence for both a one-dimensional (distress) and a two-dimensional (anxiety and depression) solution for the HSCL-10, but only a one-dimensional (distress) solution for the PADQ. The HSCL-10 and PADQ found to be moderately to highly correlated (r = 0.62, p < 0.0001, 0.73 after correction for attenuation).

**Conclusion:**

HSCL-10 has shown good screening abilities in a rural setting in Pakistan, and moderate to good concordance with an indigenous instrument measuring psychological distress. The HSCL-10 can therefore be used as a screening instrument, both in clinical and epidemiological settings in Pakistan, and for Pakistani immigrants living in Western societies.

## 1. Background

World Health Organization has predicted that by the year 2020 depression and anxiety will be the most common causes of disability in the developing world [1]. Despite this prediction, mental health services in many developing countries are not yet prepared to combat the emerging challenge. In Pakistan, for example, there are 140.7 million inhabitants but currently only 342 professionally trained psychiatrists. In 2001 only 1% of Pakistan's GDP was spent on health, and less than 1% of that on mental health services [2]. Yet this evident lack of resources has not mired the determination of mental health researchers in Pakistan. Within a short period of time they have not only sought to report the prevalence of common mental health disorders and their associated risk factors, but they have also shown enthusiasm for the development and validation of different screening instruments to identify mental health problems in Pakistan.

A review study conducted recently in Pakistan by Mirza and Jenkins (2004) reported a prevalence rate of 34% for anxiety and depressive disorders. While reporting this prevalence, however, the authors expressed their concern regarding the considerable variation in methods and instrument used across studies included in the review. The authors of the review also note, that this reported rate of prevalence in Pakistan, is comparable to the prevalence rate (21% to 57%) for the same disorders estimated in India among patients attending primary care services [3]. However, it is at present uncertain whether these high prevalence rates reflect actual levels of distress in the population or whether they reflect measuring bias due to the use of Western instruments in culturally different populations. Thus, psychometric work is crucial to determine whether potentially a majority of the Pakistani population actually suffers from common mental health problems.

According to the prevailing information from the mental health studies conducted in Pakistan, the most important commonly used mental health screening instruments in Pakistan were: Bradford Somatic Inventory (BSI) [4-10], Self-Reporting Questionnaire (SRQ) [11-17], General Health Questionnaire (GHQ) [18,19], Personal Health Questionnaire (PHQ) [11] and Hospital Anxiety and Depression Scale (HADS) [20]. Of these commonly used instruments only BSI was developed with a bilingual/bicultural approach, meaning that it was simultaneously developed in English and in Urdu- the native language of Pakistanis- with a focus on somatic symptoms of anxiety and depression presented by both ethnic Pakistanis and indigenous British patients [5]. All other instruments were originally adopted from the Western world.

During this adopting process, attention was paid not only to the translation of language, but also to the instruments' validation within Pakistan. Selected and various population groups were used in the validation studies. For example, GHQ and SRQ were validated in primary care settings in Pakistan, using clinical interviews based on Patient Assessment Schedule. Despite these attempts at validation, population based studies suggested that although some of these instruments were well suited to literate and well-educated subjects, their content and format were less appropriate for general use in Pakistan [21].

What is currently lacking from the Pakistani mental health literature is a study that compares the screening ability of any Western instrument measuring anxiety and depression with that of an indigenous scale measuring the same construct. There is currently no examination of whether the symptoms of the common mental disorders anxiety and depression are structured similarly in the Pakistani general population as it is in the Western populations. In other words, little is known about whether the Pakistani general population responds to a mental health screening instrument in a way that is comparable to the way it is responded to in western industrialized countries. There are neither conducted appropriate comparisons of western and indigenous screening instruments for symptoms of common mental disorders in Pakistan. By conducting such a study, one could gain an impression of differences or similarities in the functional ability of the two approaches to measure anxiety and depression in Eastern and in Western contexts. This information would make it possible in future to compare rates of anxiety and depression in Pakistan and the West.

The Pakistan Anxiety and Depression Questionnaire (PADQ) is one of the screening instruments that has been recently developed and validated in Pakistan. This instrument has been developed with an aim to capture the cultural related anxiety and depression symptoms within general clinical settings in Pakistan. Detailed information on its development and validation could be found elsewhere [21]. After the Agha Khan University Anxiety and Depression Scale (AKUADS)[22], the PADQ is the second instrument of its kind that has ever been developed in Pakistan in Urdu.

From the perspective of Western mental health studies, Hopkins Symptoms Checklist (HSCL) is one of the screening instruments that is well-known and widely used in epidemiological studies, with a history dating back to the 1950s s [23,24]. Several versions of the scale with differing lengths (5–90 items) have been used across a wide range of settings. In general, the reliability and validity of the HSCL measures have been well supported [25-27]. Moreover, its use has been reported in some studies screening the mental health status of some of the immigrant groups and displaced persons in Europe [28-31].

To the best of our knowledge, HSCL has never been used in Pakistan. The use of this measure in the present study thus provided us with an opportunity to investigate the HSCL-10's overall functional ability as a screening instrument for Pakistani population in comparison to the PADQ, indigenously developed and validated against ICD-10 Diagnostic Criteria in clinical settings.

Hence, the aims of the present study were to examine the psychometric properties of the HSCL-10 in a Pakistani rural normal population, and to compare it with the anxiety/depression scale (AD-scale) of the PADQ. This was done through: 1) comparison of scale and item statistics for HSCL-10 and PADQ (scale reliability, mean item score, corrected item-total correlation, and alpha if item deleted); 2) exploratory factor analyses separately for HSCL-10 and PADQ, to determine whether the instruments represent one single latent construct (distress) or two separate but correlated latent constructs (anxiety and depression); 3) investigation of concordance between HSCL-10 and PADQ for the whole sample as well as per economical strata.

## 2. Methods

### 2.1 Study design

This was a cross-sectional study employing a strategic, convenient sampling technique. Due to common illiteracy in the Pakistani rural population, the questionnaires were presented for participants in interview form.

### 2.2 Sample

Participants were recruited from six villages in the district of Gujarat in Pakistan. The names of the villages were: Mehmand Chak, Mundir Sharif, Dilu Sharif, Dara- Alam Pur Gondalan, Jundawala, and Buder murjan. These villages were selected because more than 90% of Pakistani immigrants to Norway have come from these villages. Moreover, information secured from these villages would be used to compare the mental health status of their counterparts living in Oslo, Norway, in the second phase of this study.

Geographically, these villages did not share the common boundaries and they were spread in the different directions. Therefore, to collect data from these villages at a same time with similar data collecting technique was a difficult task. We tried to get a randomized sampling but realized that within allocated resources and in the absence of any suitable account of the population in those villages it would be difficult to maintain the randomised nature of the sampling. A suitable alternative, to work within available resources and provided working condition, was to adopt a strategy that could be feasible and similar in all the villages of our interest, despite different socio-political conditions. After discussing these issues with the local health authorities and with the opinion leaders of the local community, it was decided that medical camps would be suitable alternative to recruit the participants for the study. Inclusion criteria to attend the camps and to participate in the study were that one should be resident of those villages and should be over 30 years of age. A comprehensive information campaign was run to inform the residents of these villages about the aims and objectives of the study to secure their confidence and response. Further, it was decided that participants would be provided with medicines if a need was detected. The participants were interviewed by the field workers before every participant met with the principal investigator of the study, who was a medical doctor, for consultation. Appropriate medical help was provided at camps and those who were seriously ill were referred to their local health authorities for further treatment and follow-up.

In total eighteen camps were organised in the six participating villages and the total number of participants recruited was 1040. According to local authorities total population of the selected villages as per a census conducted in 1998 was, 10 845 (record can be provided on request). Information regarding the age distribution of the population residing in these villages was not available. However, information provided did indicate that 9.5% of the total population of these villages responded to our invitation. Such a response rate can perpetuate a risk for selection bias. In particular, the data collection technique and the inherent composition of the medical camps might have attracted those people who were anxious about their health and belonged to lower socio-economic stratum and had a desire to come in contact with any physician. This issue will be addressed in the next sections of this article.

### 2.3 Data sampling procedure

From institutions providing basic health care in the villages of interest, eight health workers (male/female) were selected as field assistants. Prior to the data collection phase of the study, these field assistants were informed about the purpose of the study and were trained with respect to the questionnaire and data registration technique. All the field workers were familiar with both Urdu, the main written language of the Pakistan, and Punjabi, which was the local dialect.

The local health authorities were also informed and permission was obtained from them to conduct the survey. The field workers secured verbal consent from all the participants in the study before conducting their interviews. Participants were also consulted individually by the principal investigator for the purposes of clinical examination and blood sampling.

### 2.4 Questionnaire

The questionnaire was compiled in Urdu, the native language of Pakistanis. This questionnaire was used to collect socio-demographic information including age, sex, education, marital status, income and number of family members. The self-reported health was registered against three response categories: poor, good and very good. Two instruments the HSCL-10 and PADQ (AD- scale) were also included in the questionnaire to obtain information regarding the anxiety and depression level of participants in the study.

Participants reported their age in years. This information was categorised into four age cohorts: (30–39 years, 40–49 years, 50–59 years and > 60 years) Education was reported as number of total school years and then converted into three education categories: no education, primary/secondary education, and intermediate/university education. According to Pakistani school system, primary school comprises 1–5 years of education whereas 6–10 years, 11–12 years, and over 12 years correspond to secondary, intermediate and university education respectively.

Participants reported total annual income of their families in rupees (PKR). This figure was categorized into three levels: low (no income to 100,000 PKR), medium (100,000 to 200,000 PKR) and high income (>200,000 PKR). In forming these income categories, detailed information regarding the per capita income, family structure and socio-economic conditions of the villages included in the study were taken into consideration. Marital status was recorded in three categories: married, unmarried and partner died.

Participants were also asked to report the total number of family members in their household. This variable included all the persons living in the same house and sharing the same kitchen. From the total number of reported family members, three categories were made: 1–3, 4–6 and >6 family members.

#### 2.4.1 Instruments

##### A: Hopkins symptoms checklist (HSCL-10)

The ten-item Hopkins Check List (HSCL-10) was used to measure psychological distress. HSCL-10 demonstrate good sensivity and specificity for detecting psychological symptomatology and mental distress compared with the widely used HSCL-25 [32]. The 10-items included in HSCL 10 were originally selected by a stepwise regression analysis of data on HSCL-25 collected for a study, regression done for each item on the total score [33]. It has been further documented that like HSCL-25, the 10-items checklist also tapped both anxiety and depression. Out of 10-items 4-items indicated anxiety and 6-items were related to the symptoms of depression. The correlation between original anxiety score and short version score was 0.91 and correlation between depression scores was 0.96 [34]. The Urdu version of this instrument was obtained from previous work conducted to translate the HSCL-25 in Pakistan. A full explanation of the methodology used in the translation is available elsewhere [35].

Participants were asked to response to the following items according to their experience during the previous week:

1. Suddenly scared for no reason

2. Feeling fearful

3. Faintness, dizziness, or weakness

4. Feeling tense or keyed up

5. Blaming yourself for things

6. Difficulty in falling asleep or staying asleep

7. Feeling blue

8. Feeling of worthlessness

9. Feeling everything is an effort

10. Feeling hopeless about future

Out of the 10- items described above, the first 4-items were related to anxiety and the remaining to depression. Each item was rated on a scale from 1 (not at all) to 4 (extremely). Only those participants who answered at least 5 of the 10 items were included in the analysis and data for unanswered items was imputed using the mean value of the items responded to by the participant. In this way out of total 1040 participants only 5 were not included in the present study.

##### B: Pakistan anxiety and depression questionnaire (PADQ)

The PADQ, an instrument for screening anxiety and depressive disorders, was developed and validated in Pakistan to avoid the use of translated Western instruments. This instrument was derived from common idioms used by patients to refer their psychological distress. The ICD-10 Diagnostic Criteria for Research were used to identify cases and patients' relatives acted as controls. This instrument includes an anxiety/depression scale and a depression scale, each of 15 items. Both scales demonstrated excellent validity as screening instrument for anxiety and depressive disorders respectively in clinical settings in Pakistan. The 15 items comprising the anxiety/depression scale in this instrument were included in the present study. These items were administered in Urdu, as per the original version of the PADQ. The respondents were able to understand Urdu and they opted to report their response either in Urdu or Punjabi according to their preferences. The 15-items scale describing depression (D-scale) in the PADQ was not used for comparison purposes because its contents were specifically related to depression symptomatology. The reported sensitivity, specificity, positive predictive value and negative predictive value for its anxiety/depression scale were 96%, 88%, 93% and 94% respectively [21].

The English translation of the 15 anxiety/depression items, as provided by the author, is as follows:

1. Do you think that you have some mental problems?

2. Do you feel anxious amongst a lot of people?

3. Is your mind in peace?

4. Do you worry over trivial things?

5. Has your tolerability decreased?

6. Does one idea come to your mind again and again?

7. Have you become more irritable?

8. Do you feel lazy?

9. Have you lost your self-confidence?

10. Do you get frightened?

11. Do you feel that your mind is not working?

12. Do you feel that you are being punished for something?

13. Do you sleep well at night?

14. Do you keep on thinking without any purpose all the time?

15. Do you feel that you do not understand anything?

Despite the fact that we lack information (from author of the PADQ) about the association of the above described items with regard to anxiety and depression specifically, a review was made to determine the nature of the symptoms/expressions described in each item. Two items were clearly related to anxiety: "feel anxious amongst a lot of people" and "get frightened". Seven items were related to depression; "worry over trivial things", "more irritable", "feel lazy", "punished for something", "lost your self-confidence", "does not sleep at night" and "mind does not work". The rest of the items were not specifically related to anxiety or depression. Each item was rated dichotomously as 0 (no) and 1 (yes). Only those participants who responded at least 8 out of 15 items were included in the study. In this way out of total 1040 participants only 5 respondents were not included in the study. A threshold 5/6 was used to identify the participants with anxiety/depression.

### 2.5 Statistical analysis

The Statistical Package for the Social Sciences 14.0 (SPSS for Window 14.0) was used for statistical analysis. The internal consistencies of HSCL-10 and PADQ were measured using Cronbach's alpha coefficient, as were item analyses for both measures.

Exploratory factor analyses were performed for HSCL-10 and PADQ separately, utilising a principal axis factor extraction and Promax rotation to examine the underlying factor structure. As recommended Fabrigar et al. [36], the number of factors was specified in accordance with the hypothesised structure of measure. One factor (distress) and two factor (anxiety and depression) solutions were examined for each scale. Because the factors of anxiety and depression are likely to correlate, an oblique rotation (Promax) was selected for the two factor solutions. Pearson's correlation was used to establish correlation between the two instruments. The correlation corrected for attenuation was calculated by dividing the observed correlation by the square root of the product of the two scale reliabilities [37].

## Results

The mean age of participants was 51 years (SD 13.2) and ranged from 30 to 90 years. 32.1% of the respondents were 60 or more years of age. Overall, 63% of the participants were female and 94.3% of the participants were "married". Of those participants who reported their income, 65.5% belonged to the lower income stratum. The overall education level of the participants was poor; 41.4% of the participants were without any education whereas 32.2% reported only primary or secondary education. 82.3% of participants reported more than 4 family members compared to 16.9% who reported families composed of 1–3 members only (Table 1).

**Table 1:** Socio-demographic characteristics of the sample population (n = 1040)

**Variable**	**Number**	**Percentage**
**Age**		
30–39 years	211	20.3
40–49 years	258	24.8
50–59 years	237	22.8
60 & over	334	32.1
**Gender**		
Men	385	37.0
Women	665	63.0
**Marital status**		
Married	981	94.3
Unmarried	50	4.8
Partner died	9	0.9
**Education**		
No education	431	41.4
Primary/secondary	337	32.4
Intermediate & higher	40	3.8
Missing	232	22.3
**Family members**		
1–3 members	176	16.9
4–6 members	366	35.2
>6 members	490	47.1
Missing	8	0.8
**Yearly income**		
Low	682	65.5
Medium	183	17.6
High	129	12.4
Missing	46	4.4

The mean distress score for the HSCL-10 was 1.29 (95% CI, 1.27–1.32) and for the PADQ it was 0.20 (95% CI, 0.19–0.21). In stratified analysis, the distribution pattern of mean distress scores for the HSCL-10 and PADQ respectively were similar. An increasing trend in distress score was associated with an increase in education and with increasing income, for both instruments. Additional information about the distribution pattern of mean distress score can be seen in Table 2.

**Table 2:** PADQ and HSCL-10 scale scores and 95% confidence interval in relation to age, gender, education, marital status, family members and income

**Variable**	**PADQ**		**HSCL-10**	
	**Mean**	**95% CI**	**Mean**	**95% CI**

**Age**				
30–39 years	0.18	0.15–0.21	1.29	1.23–1.35
40–49 years	0.16	0.13–0.18	1.22	1.17–1.28
50–59 years	0.20	0.17–0.23	1.29	1.24–1.35
>60 years	0.20	0.18–0.23	1.30	1.25–1.34
		p = 0.023		p = 0.128
				
**Gender**				
Men	0.18	0.15–0.20	1.26	1.22–1.31
Women	0.19	0.18–0.21	1.29	1.26–1.33
		p = 0.186		p = 0.272
				
**Marital status†**				
Married	0.22	0.19–0.24	1.38	1.32–1.43
Unmarried	0.28	0.22–0.35	1.45	1.30–1.59
Partner died	0.24	0.09–0.39	1.51	1.20–1.82
		p = 0.142		p = 0.427
				
**Education†**				
No education	0.22	0.16–0.27	1.38	1.26–1.50
Primary/secondary	0.26	0.20–0.32	1.41	1.29–1.53
Intermediate & higher	0.27	0.18–0.35	1.54	1.37–1.72
		p = 0.016		p = 0.094
				
**Family members**				
1–3 members	0.18	0.15–0.21	1.24	1.17–1.30
4–6 members	0.18	0.15–0.20	1.28	1.23–1.32
>6 members	0.20	0.18–0.22	1.32	1.28–1.35
		p = 0.145		p = 0.080
				
**Yearly income†**				
Low	0.21	0.15–0.26	1.38	1.26–1.50
Medium	0.22	0.16–0.29	1.37	1.24–1.51
High	0.32	0.25–0.38	1.58	1.45–1.72
		p = 0.000		p = 0.000

†Marital status, education and yearly income is presented as controlled scores for age and gender with corresponding p values for differences between groups

Cronbach's alpha coefficient was calculated for the HSCL-10 (α = 0.86) and the PADQ (α = 0.84), with both indicating high internal consistency. Item means were calculated for items measuring distress on both instruments (table 3). The HSCL-10 item with the highest mean score on a scale of 1–4 was "Feeling tensed or keyed up" (mean 1.60, SD 0.80). The lowest scoring item was "Blaming yourself for things" (mean 1.13, SD 0.43. For the PADQ items, the highest mean score on a scale of 0–1 was "mind in peace" (mean 0.69 SD 0.46). The lowest scoring item was "punished for something" (mean 0.05 SD 0.22). Mean scores of all items are shown in Table 4.

**Table 3:** HSCL-10 item level values, item-total correlations, and Cronbach's alpha (N = 1040)

**Items**			**Cronbach's alpha = 0.863**	
	
	**Mean**	**SD**	**Corrected item-total correlation**	**Alpha if item deleted**
Suddenly scared for no reason	1.16	0.49	0.57	0.85
Feeling fearful	1.16	0.45	0.56	0.85
Faintness, dizziness, or weakness	1.49	0.81	0.59	0.85
Feeling tense or keyed up	1.60	0.80	0.56	0.85
Blaming yourself for things	1.13	0.43	0.54	0.86
Difficulty in falling asleep or staying asleep	1.48	0.81	0.54	0.86
Feeling blue	1.24	0.56	0.69	0.84
Feeling of worthlessness	1.16	0.48	0.62	0.85
Feeling everything is an effort	1.20	0.52	0.66	0.84
Feeling hopeless about future	1.25	0.62	0.62	0.85

**Table 4:** PADQ item level values, item-total correlations, and Cronbach's alpha (N = 1040)

**Items**			**Cronbach's alpha = 0.842**	
	
	**Mean**	**SD**	**Corrected item-total correlation**	**Alpha if item deleted**
Do you think you have some mental problem?	0.10	0.30	0.40	0.84
Do you feel anxious amongst a lot of people?	0.11	0.31	0.50	0.83
Is your mind in peace?	0.69	0.46	-0.27	0.88
Do you worry over trivial things?	0.21	0.41	0.65	0.82
Has your tolerability decreases?	0.16	0.37	0.63	0.82
Does one idea come to your mind again and again?	0.17	0.38	0.67	0.82
Have you become more irritable?	0.24	0.43	0.57	0.83
Do you feel lazy?	0.34	0.47	0.52	0.83
Have you lost your self-confidence?	0.08	0.28	0.59	0.83
Do you get frightened?	0.11	0.31	0.56	0.83
Do you feel that your mind is not working?	0.07	0.25	0.51	0.83
Do you feel that you are being punished for something?	0.05	0.22	0.45	0.84
Do you sleep well at night?	0.25	0.44	0.54	0.83
Do you keep thinking without any purpose all the time?	0.17	0.37	0.62	0.82
Do you feel that you do not understand anything?	0.08	0.27	0.58	0.83

Mean inter-item correlation ranged from 0.26 to 0.70 for the HSCL-10 and from -0.30 to 0.61 for the PADQ. The HSCL-10 corrected item-total correlations ranged from 0.54 (blaming yourself, difficulty in sleep) to 0.69 (feeling blue). Alphas when a particular item was deleted ranged from 0.84 (feeling blue, everything is an effort) to 0.86 (blaming yourself, difficulty in sleep) in contrast to the to the computed scale alpha of 0.86 (See Table 3). Correspondingly, PADQ corrected item-total correlations ranged from -0.27 (mind in peace) to 0.65 (worry over trivial things). Alphas when a particular item was deleted ranged from 0.82 (worry over trivial things, decreased tolerability, idea in mind) to 0.88 (mind in peace), contrasted with the computed scale alpha of 0.84 (See Table 4).

The Kaiser-Meyer-Olkin (KMO) measure of sampling adequacy and Bartlett Test of Sphericity (BTS) were conducted prior to factor extraction to determine if the data were suitable for factor analysis. For HSCL-10, KMO analysis produced an index of 0.87, and BTS was highly significant (χ^2 ^[df = 190] = 4512, p < 0.0001) indicating that data were appropriate for factor analysis. Likewise, for PADQ, KMO analysis produced an index of 0.91 and BTS was also significant (χ^2 ^[df = 105] = 5638, p < 0.0001).

One factor (distress) solutions accounted for 48% of the variance in the HSCL-10, and for 39% of the variance in the PADQ. The screeplot indicated one major factor for both scales, with eigenvalue of 4.78 for the HSCL-10 and 5.89 for the PADQ. Tables 5 and 6 show factor loading for the identified factors, one and two factor solution for HSCL-10 and one- and two-factor solution for PADQ respectively. The one-factor solution- distress- yielded factor loadings ranging from 0.55 to 0.77 for the HSCL-10. The item "mind in peace" showed low factor loading (-0.29) for the PADQ. Apart from this item, all other items of the PADQ loaded in the range of 0.45 to 0.71. A two-factor solution for the HSCL-10- anxiety and depression- accounted for 60% of the variance, the second factor with an eigenvalue of 1.2. The factors correlated 0.62. Four anxiety related items with factor loadings ranging from 0.46 to 0.81, and six depression related items with factor loadings ranging from 0.24 to 0.85 were identified. For the PADQ, a two-factor solution accounted for 49% of the variance, the second factor with an eigenvalue of 1.4. The factors correlated 0.65. No clear pattern of factor loading was seen for anxiety and depression in the PADQ (See table 5 &6)

**Table 5:** HSCL-10 item-factor loading

**Items**	**Factor loading**		
	
	**One factor solution**	**Two factor solution**	**Two factor solution**
	
	**Distress**	**Anxiety**	**Depression**
Suddenly scared for no reason	0.63	**0.81**	-0.08
Feeling fearful	0.61	**0.81**	-0.10
Faintness, dizziness, or weakness	0.60	**0.57**	0.10
Feeling tense or keyed up	0.56	**0.46**	0.16
Blaming yourself for things	0.59	**0.35**	**0.30**
Difficulty in falling asleep or staying asleep	0.55	**0.37**	**0.24**
Feeling blue	0.77	**0.28**	**0.55**
Feeling of worthlessness	0.70	-0.06	**0.84**
Feeling everything is an effort	0.74	-0.03	**0.85**
Feeling hopeless about future	0.71	-0.01	**0.79**

**Table 6:** PADQ item-factor loading

**Items**	**Factor loading**		
	
	**One factor solution**	**Two factor solution**	**Two factor solution**
	
	**Distress**	**Factor 1**	**Factor 2**
Do you think you have some mental problem?	0.45	**0.48**	0.04
Do you feel anxious amongst a lot of people?	0.56	**0.38**	**0.24**
Is your mind in peace?	-0.29	**0.12**	**0.19**
Do you worry over trivial things?	0.68	-0.05	**0.77**
Has your tolerability decreases?	0.67	-0.01	**0.73**
Does one idea come to your mind again and again?	0.71	-0.05	**0.81**
Have you become more irritable?	0.63	-0.05	**0.72**
Do you feel lazy?	0.52	0.05	**0.51**
Have you lost your self-confidence?	0.65	**0.45**	**0.27**
Do you get frightened?	0.62	**0.40**	**0.29**
Do you feel that your mind is not working?	0.58	**0.73**	-0.04
Do you feel that you are being punished for something?	0.51	**0.75**	-0.13
Do you sleep well at night?	0.58	0.04	**0.58**
Do you keep thinking without any purpose all the time?	0.67	0.20	**0.53**
Do you feel that you do not understand anything?	0.64	**0.74**	0.02

Pearson's correlation coefficient was computed for sum scores of the HSCL-10 and PADQ (*r *= 0.62, p < 0.0001, 0.73 after correction for attenuation). This result indicated a positive association of moderate strength between the two scales, such that individuals who scored higher on the HSCL-10 also tended to score higher on PADQ. In order to examine the effects of potential selection bias with regard to low SES and high levels of somatic symptoms, correlations were also run in sub-samples. When stratified by income, the correlations were as follows: missing (n = 46) *r *= 0.35 (p = 0.005); low (n = 676) *r *= 0.62 (p < 0.0001); medium (n = 183) *r *= 0.59 (p < 0.0001); and high (n = 129) *r *= 0.72 (p < 0.0001). When stratified by self-perceived somatic health, correlation for those who had not responded (n = 58) *r *= 0.63 (p < 0.0001); for those reporting this "poor" (n = 582) *r *= 0.62 (p < 0.0001); for those reporting this to be "good/very good" (n = 339) r = 0.53 (p < 0.0001).

Moreover, bivariate analysis showed that out of those who were diagnosed for psychological distress by PADQ, only 35% were also identified by HSCL-10 at conventional cut-off score (1.85) and 65% were discordant cases. A better concordance was observed between these two instruments by making a reduction in the cut-off score of HSCL-10. See table 7 for detailed description of concordance and discordance between these two instruments at different cut-off scores of HSCL-10.

**Table 7:** Correspondence between HSCL-10 at different cut-offs and PADQ

	**PADQ**	
	
**HSCL-10 (1.85)**	No diagnosis	Diagnosis
	
No diagnosis	87.4% (95.2%)	12.6% (65.0%)
Diagnosis	39.4% (4.8%)	60.6% (35.0%)
		
**HSCL-10 (1.75)**		
No diagnosis	89.1% (93.7%)	10.9% (54.4%)
Diagnosis	39.7% (6.3%)	60.3% (45.6%)
		
**HSCL-10 (1.65)**		
No diagnosis	90.2% (91.9%)	9.8% (47.2%)
Diagnosis	42.1% (8.1%)	57.9% (52.8%)
		
**HSCL-10 (1.55)**		
No diagnosis	91.6% (88.4%)	8.4% (38.3%)
Diagnosis	47.1% (11.6%)	52.9% (61.7%)
		
**HSCL-10 (1.45)**		
No diagnosis	93.2% (85.4%)	6.8% (29.4%)
Diagnosis	49.4% (14.6%)	50.6% (70.6%)

• Percentages in parentheses are column percentages

• Both column and row percentages are equal to 100

## Discussion

The present study compared the psychometric properties and concordance between two anxiety/depression instruments- the HSCL-10 and AD-scale of the PADQ- in a Pakistani rural community sample. Overall, both instruments seem to be adequate screening tools for anxiety/depression and show concordance acceptable to the conventional standards.

All the HSCL-10 inter-item correlations and corrected item-total correlations demonstrated that no single item deviated in any significant way from the overall scale functioning. This was not true, however, for the PADQ, wherein "mental problem" and "mind in peace", were inadequately correlated to other items. In contrast to other items, which describe specific symptoms of anxiety and depression, the "mental problems" and "peace in mind" items are of general nature, and may indicate an underlying construct such as "mental illness". Prejudices against mental illness in the population, conceived of as a state of arrested or incomplete development of the mind which results in impaired intelligence and social functioning, and often aggressive or seriously irresponsible conduct, may explain the low correlation to the other items [2].

Factor analyses were preformed to examine whether the HSCL-10 and the PADQ should be perceived as one dimensional (distress) or two dimensional (anxiety and depression) measures. One factor solutions accounted for 48% and 39% of the variance in the HSCL-10 and PADQ, respectively. In these solutions, all items on both scales (with the exception of "mind in peace" in the PADQ) seemed to load relatively evenly on the distress-dimension.

The two-factor solution with oblique rotation accounted for 60% and 49% of the variance in the HSCL-10 and PADQ, respectively. Whereas the factor loadings for some items on both scales increased, several factor loadings decreased. This indicates that the HSCL-items "blaming yourself", "difficulty in sleep" and "feeling blue" account for variance in both anxiety and depression, and thus do not seem to differentiate between these two conditions. The same seems to be the case with some of the items from the PADQ: "anxious amongst a lot of people", "lost your self-confidence", "get frightened" and "mind in peace".

The two-factor solution for HSCL-10, besides overlapping of factor loadings for items described above, did show a clustering pattern in factor loadings both for anxiety and depression related items, respectively. The two-factor solution for PADQ, however, showed a less clear pattern. Whereas the two anxiety related items ("anxious among a lot of people" and "get frightened") loaded on both factors, the same was true for one of the depression related items ("lost your self-confidence"). For the rest of the depression related items, there was not clear pattern on which factor they loaded.

From these analyses it could be concluded that both the HSCL-10 and PADQ may be meaningfully interpreted as a one-dimensional measure of distress. However, the HSCL-10 could also be interpreted as a two-dimensional measure because of the emerging pattern of clustered factor loadings for both anxiety and depression. A two- dimensional interpretation of the PADQ was not evident.

The HSCL-10 and PADQ were found to be moderately to highly correlated (0.62, 0.73 after correction for attenuation). Although this level of correlation is acceptable when comparing psychological instruments, a great deal of variance remains unexplained. It is proposed that problems comparing the measurement of psychiatric morbidity across the two screening instruments may have arisen from differences in the constructs they use. It appears that the construct used for anxiety and depression in the HSCL-10 was suitable for general population in Pakistan. This observation is in accordance with other studies describing the HSCL as a valid and effective screening tool for use across clinical [38], non-clinical and in community settings [29,39] as well as from a cross- cultural perspective [40].

In contrast, some items of the PADQ seem to be more valid in the clinical settings, where subjects define themselves as psychiatric patients. This might be due to a difference between psychiatric symptom constellations that exist in a particular community to those experienced in hospitals, which are related to define certain diagnosis [41]. This is further supported by the fact that high clinical validity for an instrument defined and tested in a patient population has often not been achieved in field studies in general populations [42]. Moreover, previous studies support this assertion and report that difficulties arise when one instrument, developed to measure a given construct in one particular group, may not validly assess the same construct in other groups due to conceptual or metric differences [43,44].

By correspondence analysis (Table 7), 35% concordance was observed on diagnosed cases between PADQ and HSCL-10 at its conventional cut-off score 1.85 [32]. A gradual improvement was observed as we go downwards from the conventional cut-off score of HSCL-10. More than 50% concordance on diagnosed cases was achieved at 1.65 cut-off score. Previously, a study conducted by Sandanger et al. has shown 46% concordance between cases diagnosed by the Composite International Diagnostic Interview (CIDI) and cases identified by HSCL-25 at its conventional cut-off 1.75 [45]. It is however important to keep in mind that reports of the agreement between psychiatric symptom screening and medical diagnosis are seldom better than 50% [42].

An increased concordance by reducing cut-off score of HSCL-10 could be attributed to the fact that HSCL-10 is a self-inventory but in our study it was used in interview format. The use of HSCL in interview format has been reported in other studies while dealing with non-literate populations [46]. It has been generally reported that social desirability and other factors related to the interview technique can have negative impact on response patterns compared to self-reporting [47]. Sandanger et al. has discussed a possibility of lower score on HSCL-25 in general population when list of symptoms was presented orally by an interviewer, rather than the respondents checking off her/his symptoms on sheet by her/himself [45], probably due to social desirability bias [48].

Another explanation of this observation could be related to the cultural aspects linked with the expression of psychological distress. A change in the cut-off score is in accordance with other studies, suggesting a more cautious approach to the cross-cultural use of pre-determined cut-off scores [49]. A change to the optimal cut-off scores has also been observed with other well established instruments in various studies [50]. A recently conducted study in Afghanistan, a neighbour country at the northern border of Pakistan, has raised concerns over administration of self-administered questionnaires by interview format and the role of culture in defining response on mental health symptoms by the respondents [46].

Our sample was self-motivated and respondents were invited to the field camps with a message to get a free medical consultation and blood test for diabetes. Hence it might be possible that the participants were overrepresented with respect to somatic disorder and poor socio-economic status. In order to examine the effect of this sampling bias on the main findings in this article, regarding the concordance between HSCL-10 and PADQ, we calculated correlations between the sum scores of the two measures separately among people with low, middle and high income and among people with self-reported poor and good somatic health, respectively. These correlations showed a slight tendency in the direction of biased results with regard to both hypothesized sources of bias, however in opposite directions. In other words; when comparing correlation between the two scales in the different income strata, this was largest for those with the highest income (the group underrepresented in this sample). Whereas comparing correlations in the groups of people reporting somatic health problems, the correlation was highest among those with poor health (the group overrepresented in this sample). Thus, we have one effect suggesting that there is a bias decreasing the strength of our results due to sampling bias and one effect suggesting an increase. We therefore hypothesize that these opposite effects approximately equal each other so that the effect of sampling bias on our results taken together is relatively small.

In mental health studies, the term "gold standard" usually attributed to the clinical interviews and they are relatively irrefutable standards those constitute recognised and accepted evidence that a disease exists [51] and it is customary to use them in validation studies. Also in our study it would have been a better option to include clinical interviews but limited resources and available working conditions in community settings and the purpose of the study contained us to conduct this study without clinical interviews.

Another possible limitation of the present study might be due to overrepresentation of the females in our sample, 63% of the study's sample was female. Yet, we were unable to detect any bias due to this imbalance when conducted exploratory factor analysis stratified by gender (data not shown).

## Conclusion

The focus in this study was to compare the psychometric properties of a western screening tool for anxiety and depression, HSCL-10, in Pakistan and to compare it with the indigenous PADQ (AD-scale). This comparison was done to observe the construct of anxiety and depression in different cultural perspective. We find better scale-functioning in HSCL-10, and find that the two screening instruments were comparable in many respects, but not all. On the basis of observed psychometric properties and screening ability we can conclude that HSCL-10 employed in Pakistan was approximately as good as PADQ, for assessing mental health, and may therefore be used as a mental health screening tool in Pakistan and among Pakistani immigrants living in the West.

## Competing interests

The authors declare that they have no competing interests.

## Authors' contributions

HRS designed the study and wrote the protocol. HDZ contributed by his comments on the manuscript and contributed substantially in the factor analysis. OSD supervised and guided through the whole process of this study. ID was the main person helping with statistics and contributed also with her comments on the substance of the manuscript. NA also finally gave her comments on the contents of the manuscript.

## Pre-publication history

The pre-publication history for this paper can be accessed here:


